# Japanese traditional dietary fungus koji *Aspergillus oryzae* functions as a prebiotic for *Blautia coccoides* through glycosylceramide: Japanese dietary fungus koji is a new prebiotic

**DOI:** 10.1186/s40064-016-2950-6

**Published:** 2016-08-11

**Authors:** Hiroshi Hamajima, Haruka Matsunaga, Ayami Fujikawa, Tomoya Sato, Susumu Mitsutake, Teruyoshi Yanagita, Koji Nagao, Jiro Nakayama, Hiroshi Kitagaki

**Affiliations:** 1 Department of Environmental Science, Faculty of Agriculture, Saga University, Honjo-cho, Saga City, Saga Japan; 2 Department of Applied Biochemistry and Food Science, Faculty of Agriculture, Saga University, Honjo-cho, Saga City, Saga Japan; 3 Faculty of Health and Nutrition Science, Nishikyushu University, Ozaki, Kanzaki-cho, Kanzaki City, Saga Japan; 4 Department of Bioscience and Biotechnology, Faculty of Agriculture, Kyushu University, Hakozaki, Higashi-ku, Fukuoka, Japan

**Keywords:** Japanese cuisine, Koji, Glycosylceramide, Intestinal microbial flora, *Blautia*, *Aspergillus*, Prebiotic

## Abstract

**Background:**

The Japanese traditional cuisine, Washoku, considered to be responsible for increased longevity among the Japanese, comprises various foods fermented with the non-pathogenic fungus *Aspergillus oryzae* (koji). We have recently revealed that koji contains an abundant amount of glycosylceramide. Intestinal microbes have significant effect on health. However, the effects of koji glycosylceramide on intestinal microbes have not been studied.

**Materials and methods:**

Glycosylceramide was extracted and purified from koji. C57BL/6N mice were fed a diet containing 1 % purified koji glycosylceramide for 1 week. Nutritional parameters and faecal lipid constituents were analyzed. The intestinal microbial flora of mice on this diet was investigated.

**Results:**

Ingested koji glycosylceramide was neither digested by intestinal enzymes nor was it detected in the faeces, suggesting that koji glycosylceramide was digested by the intestinal microbial flora. Intestinal microbial flora that digested koji glycosylceramide had an increased ratio of *Blautia coccoides*. Stimulation of *B. coccoides* growth by pure koji glycosylceramide was confirmed in vitro.

**Conclusions:**

Koji functions as a prebiotic for *B. coccoides* through glycosylceramide. Since there are many reports of the effects of *B. coccoides* on health, an increase in intestinal *B. coccoides* by koji glycosylceramide might be the connection between Japanese cuisine, intestinal microbial flora, and longevity.

## Background

The Japanese traditional cuisine Washoku was recently registered by UNESCO as an intangible cultural heritage (United Nations Educational, Scientific and Cultural Organization 2013). A number of mechanisms linking Japanese cuisine to its health effects have been proposed, such as the preponderance of low saturated fatty acids, high fibre, and omega-3 polyunsaturated fatty acids. However, some unknown factors besides nutritional components such as protein, fat, carbohydrate balance, or dietary fibre are considered to influence the longevity of the Japanese (Yamamoto et al. 2016); a concrete mechanism linking Japanese cuisine and its health effects remains to be elucidated.

One common characteristic of Japanese cuisine is that it contains various and abundant fermented foods. Most Japanese fermented foods contain koji (rice fermented with the non-pathogenic fungus *Aspergillus oryzae* or *A. luchuensis* (Machida et al. 2005)) as the saccharifying agent of the starch contained in crops (Kitagaki and Kitamoto 2013). These include miso (soybean and barley fermented with koji), shoyu (soy sauce), amazake (rice koji beverage), osu (rice vinegar), kurosu (black rice vinegar), sake (alcoholic beverage fermented with koji), and shochu (distilled alcoholic beverage fermented with koji). Food manufactured using fungi as the saccharifier is found in countries in the east and southeast regions of Asia, such as Korean makgeolli, Chinese huangjiu, and Indonesian tempe. Since the Japanese traditional dietary fungus *A. oryzae* has been bred and maintained as a safe and non-mycotoxin-producing fungus (Machida et al. 2005) and used in the food culture in Japan (Murakami 1985) for centuries, the US Food and Drug Administration (FDA) recognizes koji as generally regarded as safe (GRAS) and the Brewing Society of Japan lists the koji-producing fungi, *A. oryzae* and *A. luchuensis*, as the “national fungi” of Japan. However, few studies report the functionalities of eating koji. One possible explanation is the fact that *Aspergillus* mycelia contain β-glucan (Ishibashi et al. 2004), which activates macrophages through Dectin-1 (Brown and Gordon 2001) and improves glycaemic index (Jenkins et al. 2002) and serum cholesterol (Wang et al. 2016). However, the nutritional benefit of eating koji or *A. oryzae* has not been studied.

In earlier studies, we have elucidated that koji contains abundant glycosylceramide (0.5–3 mg/g dry weight) (Hirata et al. 2012; Takahashi et al. 2014; Sawada et al. 2015), which is one of the highest amounts found in any cuisine. Glycosylceramide is composed of a sugar moiety, fatty acid moiety, and sphingoid base moiety, and is categorized as a sphingolipid. Sphingolipids are critical components of the cell membrane and exert various biological functions (Truman et al. 2014; Russo et al. 2013). Koji glycosylceramide consists of *N*-2′-hydroxyoctadecanoyl-l-*O*-β-d-glucopyranosyl-9-methyl-4,8-sphingadienine (69.7 %) and N-2′-hydroxyoctadecanoyl-l-*O*-β-d-galactopyranosyl-9-methyl-4,8-sphingadienine (30.3 %) (Hamajima et al. 2016). These chemical structures differ from those in other species and the Japanese have consumed koji glycosylceramide for centuries, with current consumption being 25.7–77.1 mg glycosylceramide per day (Yunoki et al. 2008). Koji glycosylceramide might exert unique effects that could contribute to the health benefits of Japanese cuisine.

Intestinal microbial flora has a great impact on health (Fukuda et al. 2011; Sommer and Bäckhed 2013; Kanauchi et al. 2013), and many diseases are reported to be related to intestinal microbial flora (Round and Mazmanian 2009; Hold 2016; Benakis et al. 2016; Del Chierico et al. 2016). Interestingly, great variations in intestinal microbial flora among several ethnic groups are observed (De Filippo et al. 2010; Moeller et al. 2014; Nakayama et al. 2015). Thus, food content is considered to affect the intestinal microbial flora, which might provide a new therapeutic strategy for diseases. However, the relationship between Japanese food and intestinal microbial flora remains unknown.

In this study, we hypothesized that koji glycosylceramide alters the intestinal microbial flora. Specifically, that koji glycosylceramide increases the content of several microbes, including *Blautia coccoides*. Since *B. coccoides* is reported to have several health benefits, an increase of *B. coccoides* through the intake of koji glycosylceramide might be one mechanism explaining Japanese longevity. This knowledge can be utilized to improve the nutritional content of foods of other nations and increase life expectancy around the world.

## Methods

### Materials

Pre-gelatinized dried koji (rice polishing ratio 70 % w/w fermented with *A. oryzae*) was purchased from Tokushima Seikiku Co., Ltd (Tokushima, Japan).

### Bacterial strains

*Blautia coccoides* (ATCC^®^ 29236; ATCC, VA, USA), *Escherichia coli* (New England Biolabs, MA, USA) and *Lactobacillus casei* (Saga University) were used in this study.

### Lipid extraction and purification

Lipid extraction from koji was performed as described earlier (Hirata et al. 2012; Takahashi et al. 2014). The lipid was extracted from 1.8 kg koji by chloroform–methanol (2:1, v/v) at a concentration of 100 mg/mL. The extracted lipid solution (10 mL) was dried by evaporator, and the dried lipids were dissolved in 5 mL of chloroform. This chloroform solution was incubated at 4 °C for 1 h and the precipitate was filtered and removed. The solution was evaporated and dissolved in chloroform–methanol (2:1, v/v) at a concentration of 100 mg/mL. The precipitate was dissolved in chloroform–methanol (2:1, v/v) as above. The solution (5 mL) was dried by evaporator and dissolved in 5 mL of acetone. This acetone solution was incubated on ice for 1 h and the precipitate and supernatant were separated by centrifugation. The precipitate was washed with cold acetone twice. The recovered supernatant and the washed acetone fraction were dried by evaporator. The dried sample was dissolved in chloroform–methanol (2:1, v/v) at a concentration of 100 mg/mL and used as the purified koji glycosylceramide in further analyses.

### Animals and diets

All aspects of the experiments were conducted according to the guidelines provided by the ethical committee for experimental animal care at Saga University. Five-week-old male C57BL/6N mice were purchased from Kyudo Co., Ltd. (Saga, Japan). The mice were individually housed in plastic cages in a temperature-controlled room (24 °C) under a 12 h light/dark cycle. The basal semisynthetic diets were prepared according to the recommendations of the AIN-76 (Shirouchi et al. 2007) (Table 1). Koji glycosylceramide was extracted and purified as above, and the general components of the samples were routinely determined according to official AOAC methods. The mice were assigned to two groups (three mice each) that were fed one of two diets (Table 1), a semisynthetic AIN-76 diet (Control group) or a semisynthetic AIN-76 diet supplemented with 1 % purified koji glycosylceramide. The mice received the diets ad libitum using Rodent CAFE (KBT Oriental Co., Ltd., Saga, Japan) for 1 week. At the end of the feeding period, the mice were sacrificed by exsanguination from the heart under isoflurane anaesthesia following a 9 h starvation period. Kidneys, adrenal glands, perirenal white adipose tissue, spleens, appendix, brains, and livers were excised immediately, and the serum was separated from the blood.

**Table 1:** Composition of experimental diets

	Nor	Kgc
Casein	20.0	20.0
Corn starch	15.0	15.0
Cellulose	5.0	5.0
Mineral mixture^a^	3.5	3.5
Vitamin mixture^a^	1.0	1.0
dl-Methionine	0.3	0.3
Choline bitartrate	0.2	0.2
Corn oil	7.0	7.0
Purified koji glycosylceramide	–	1.0
Sucrose	48.0	47.0

Nor = control and Kgc = koji-supplemented diet

^a^AIN-76

### Analysis of hepatic lipids and serum parameters

Liver lipids were extracted according to the method of Folch et al. (1957), and the concentrations of triglycerides, cholesterols, and phospholipids were measured using the methods of Fletcher (1968), Sperry and Webb (1950), and Rouser et al. (1966), respectively. The triacylglycerol, cholesterol, phospholipid, and glucose levels in the serum were measured using enzyme assay kits from Wako Pure Chemicals (Tokyo, Japan).

### Analysis of intestinal microbial flora

Faecal samples were freeze-dried for 3 days, and genomic DNA was extracted from the faecal samples using the bead-beating method as described previously (Matsuki et al. 2004). The region containing the 16S rRNA V3–V4 variable region was amplified by PCR according to the Illumina protocol (Illumina Inc. 2013), and the sequences were analyzed using Miseq (Illumina, Inc., CA, USA). The Miseq Reporter 16S metagenomics system was used to obtain the information on the bacterial composition. The microbial cluster tree was categorized using the NCBI taxonomy browser (MD, USA).

### In vitro analysis of the effect of koji glycosylceramide on microbial growth

*Blautia coccoides* was inoculated in yeast-peptone-dextrose (Becton, Dickinson and Company, NJ, USA) medium containing sodium cholate (0.0015 % v/v) and glucosylceramide (4 μg/μL in ethanol) or vector ethanol (1 % v/v) at a cell density of 1 × 10^6^ cells/mL. The bacterial cells were incubated in anaerobic jars (AnaeroPack Kenki, Mitsubishi Gas Chemical Co., Inc., Tokyo, Japan) at 30 °C for 24 h. Cultures were homogenized and the OD_600_ was measured using a spectrophotometer (UV-1800; Shimadzu, Kyoto, Japan). To incubate *Lactobacillus casei*, MRS medium (Becton, Dickinson and Company) was used; to incubate *Escherichia coli*, Luria-Bertani (LB) medium (Nissui, Tokyo, Japan) was used.

### Preparation of intestinal extracts

Fresh mouse small intestine was thoroughly washed with washing buffer (50 mM Tris-HCl buffer pH 7.5, 150 mM NaCl). The small intestine was cut into small pieces and collected in a clean plastic tube. The collected fragments of small intestine were homogenized with the Polytron 10/35 (Kinematica Inc., Luzern, Switzerland) in homogenization buffer (50 mM Tris-HCl buffer pH 7.5, 150 mM NaCl, protease inhibitor cocktail set V, and 1 mM phenylmethylsulfonyl fluoride; Wako Pure Chemicals, Tokyo, Japan). The homogenate was centrifuged at 500×*g* for 5 min to remove debris. The protein concentration of the supernatant was determined using a DC protein assay (Bio-rad Laboratories, Inc., CA, USA).

### Activity assay of intestinal enzymes

The purified koji glycosylceramide (0.5 or 1.0 mg) was dissolved in reaction buffer (50 mM Tris-HCl buffer pH 7.5, 0.5 % w/v Triton X-100). Intestinal extract corresponding to 2 mg protein diluted twofold with pure water was added to the solution. The reaction solution was incubated at 37 °C for 18 h or 30 h. The reaction was stopped by boiling for 5 min, and then the solution was freeze-dried. The samples were dissolved in chloroform–methanol (2:1, v/v) and applied to TLC analysis. TLC was developed with chloroform–methanol–acetic acid–water (20:3.5:2.3:0.7, v/v). Detection was performed with 2 mg/mL orcinol in 70 % H_2_SO_4_ reagent. Ceramidase activity was measured using C12-NBD-ceramide (Avanti Polar Lipids, Inc., AL, USA) as the substrate (Mitsutake et al. 2001). C12-NBD-ceramide (1 nmol) was incubated at 37 °C for 18 h with 0.5 mg of the intestine extract. Chloroform–methanol (2:1, v/v) was added to the reaction mixture, the lower phase was collected and applied to a TLC analysis plate and developed with chloroform–methanol–25 % ammonia (90:20:0.5, v/v) and visualized by fluorescence.

## Results

### Extraction and purification of koji glycosylceramide

**Fig. 1:**
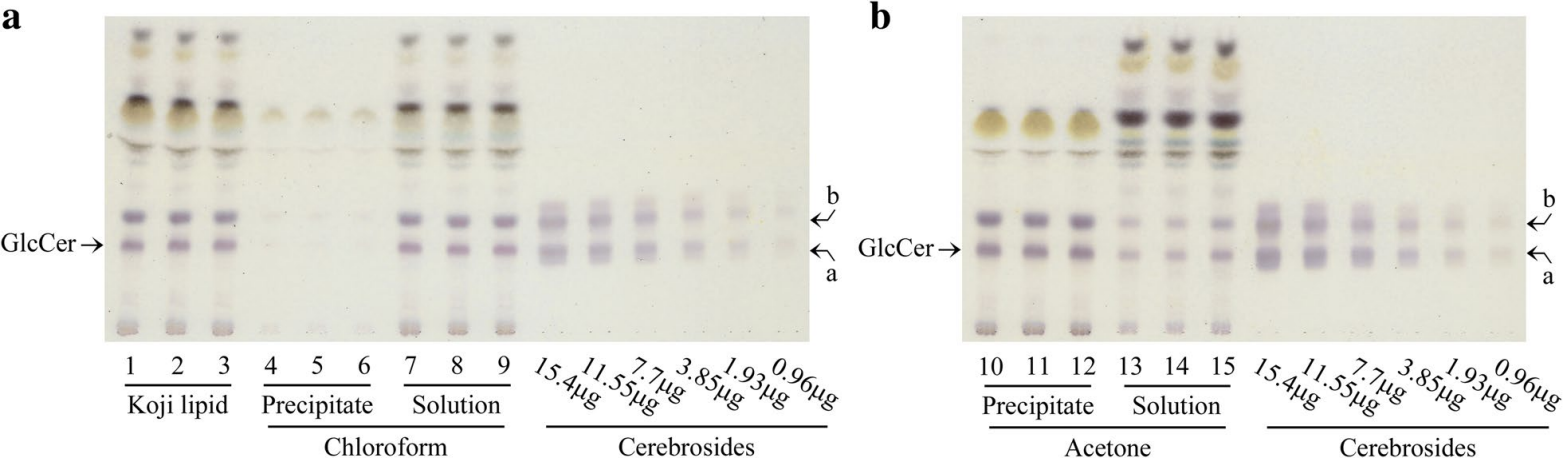
Extraction and purification of glycosylceramide from koji. Lipids were extracted with chloroform–methanol from 1.8 kg of pregelatinized koji. The ester-bond containing lipids were degraded with mild alkaline treatment, and the lipid phase was extracted using Bligh and Dyer fractionation. The chloroform-soluble fraction was recovered (**a**), and the acetone-insoluble fraction was recovered **(b)**. GlcCer indicates glycosylceramide, *a* and *b* indicate hydroxylated and nonhydroxylated cerebroside respectively

First, glycosylceramide was extracted and purified from 1.8 kg koji. Lipids were extracted with chloroform–methanol, and ester-linked lipids were degraded by mild alkaline treatment. Glycosylceramide was further purified with chloroform–acetone fractionation (Fig. 1a, b). Table 2 shows the result of purification of glycosylceramide from 1.0 g koji. Finally, 2.8 g of koji glycosylceramide was purified as a major band containing two minor bands (Fig. 1b).

**Table 2:** Purification summary of glycosylceramide from koji

	Koji lipid	Chloroform		Acetone	
		Soluble fraction	Insoluble fraction	Insoluble fraction	Soluble fraction
Weight of total recovery		0.39 g ± 0.01	0.59 g ± 0.01	0.25 g ± 0.04	0.24 g ± 0.06
Weight of glycosylceramide	24.49 mg ± 2.49	0.17 mg ± 0.02	22.24 mg ± 0.84	17.80 mg ± 1.63	3.64 mg ± 2.36
Purification rate of glycosylceramide	2.46 % ± 0.21	0.04 % ± 0.00	3.79 % ± 0.18	7.28 % ± 0.59	1.41 % ± 0.56

Summary of glycosylceramide purification from 1 g of koji lipid. Glycosylceramide was purified from koji by chloroform–acetone fractionation

The results are expressed as mean values ± standard deviation of three independent experiments

### Feeding of koji glycosylceramide

Purified koji glycosylceramide was fed to mice for 1 week (Table 2). Although the changes were not statistically significant (*p* > 0.05), the levels of serum and liver triglycerides increased while those of serum glucose and liver cholesterol decreased (Table 3). Additionally, the relative weight of the adrenal gland increased while the relative weight of the perirenal white adipose tissue decreased (Table 4). These results suggest that fed glycosylceramide was metabolized to triglyceride in the intestine and appears to play a role in decreasing serum glucose levels while increasing the relative weight of the adrenal gland. The precise meaning of these changes awaits further analysis.

**Table 3:** Effect of koji glycosylceramide on serum and hepatic parameters in mice

	Nor	Kgc
Serum		
Cholesterol (mg/dL)	101 ± 2	96.0 ± 1.4
Triglyceride (mg/dL)	67.6 ± 0.7	104 ± 14
Phospholipid (mg/dL)	207 ± 1	213 ± 7
Glucose (mg/dL)	248 ± 2	224 ± 7
Liver		
Cholesterol (mg/g liver)	3.03 ± 0.05	2.60 ± 0.16
Triglyceride (mg/g liver)	23.6 ± 5.0	26.3 ± 4.5
Phospholipid (mg/g liver)	33.4 ± 5.1	30.0 ± 1.1

“Nor” = control and “Kgc” = koji-supplemented diet

**Table 4:** Effects of koji glycosylceramide on growth parameters in mice

	Nor	Kgc
Initial body weight (g)	18.9 ± 0.6	18.9 ± 0.5
Final body weight (g)	20.0 ± 0.7	19.8 ± 0.3
Body weight gain (g)	1.13 ± 0.16	0.900 ± 0.208
Food intake (g)	19.2 ± 1.0	20.4 ± 1.1
Food efficiency (g)	0.0603 ± 0.0122	0.0441 ± 0.0098
Liver (g/100 g B.W.)	5.78 ± 0.08	6.44 ± 0.18
Kidney (g/100 g B.W.)	1.33 ± 0.05	1.38 ± 0.01
Adrenal gland (g/100 g B.W.)	0.0216 ± 0.0009	0.0352 ± 0.0101
Perirenal white adipose tissue (g/100 g B.W.)	0.605 ± 0.052	0.424 ± 0.058
Spleen (g/100 g B.W.)	0.277 ± 0.021	0.289 ± 0.014
Appendix (g/100 g B.W.)	0.977 ± 0.063	1.14 ± 0.05
Brain (g/100 g B.W.)	2.01 ± 0.12	2.06 ± 0.08

B.W.: Body Weight

“Nor” = control and “Kgc” = koji-supplemented diet

### Metabolic fate of koji glycosylceramide during passage through the intestine

**Fig. 2:**
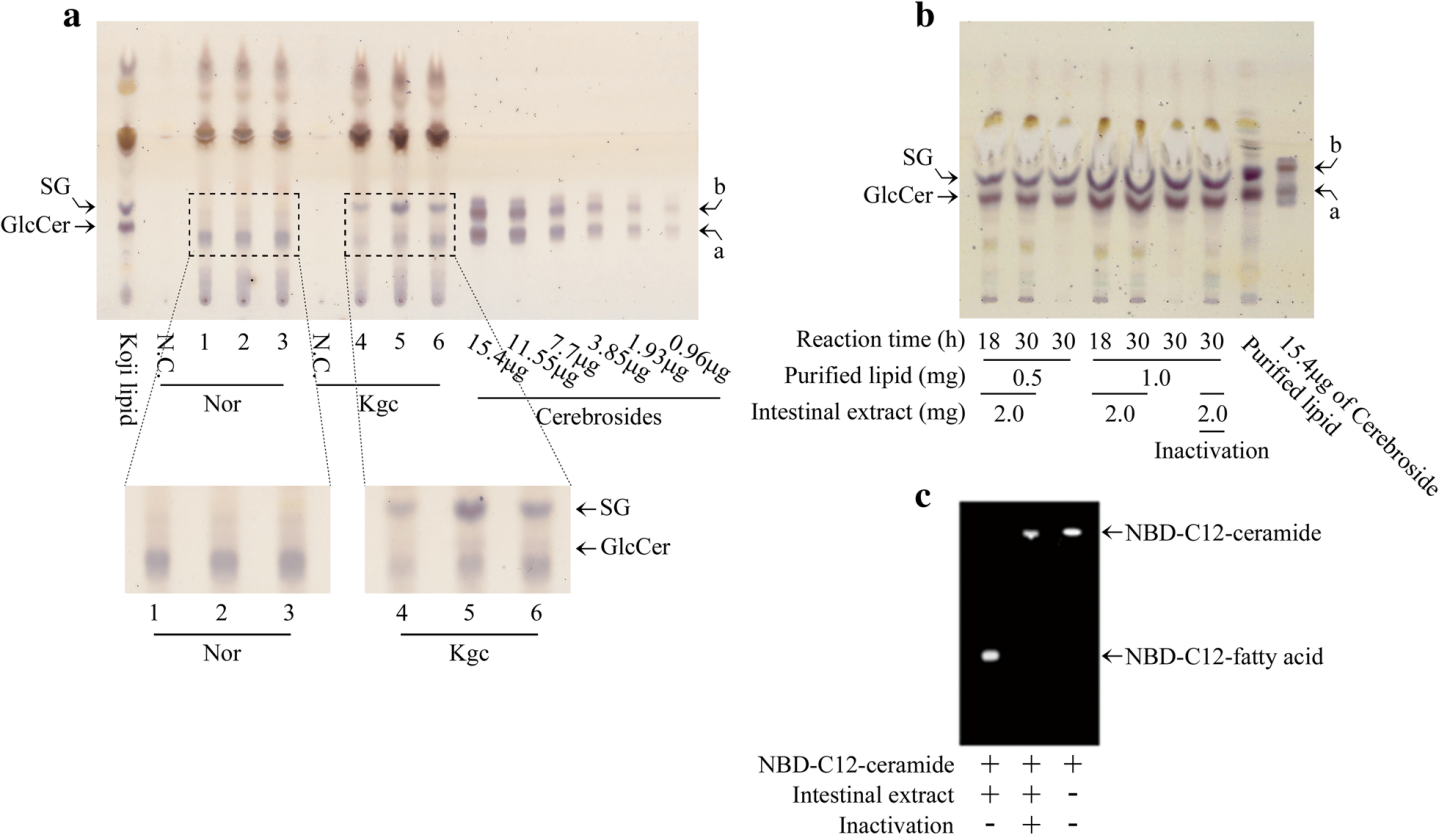
Metabolic fate of koji glycosylceramide in the intestine. **a** Total lipid profile of feces from mice fed with or without koji glycosylceramide. Nor indicates the feces of non-added mice and Kgc indicates those of koji glycosylceramide-fed mice. The *number* indicates the replicate number of experiments. **b** Total lipid profile of koji glycosylceramide incubated with intestinal extracts. **c** NBD-TLC of C12-ceramide incubated with intestinal extracts. Intestinal extract was recovered from mice, mixed with purified koji glycosylceramide or NBD-C12-ceramide, incubated at 37 °C for 16–30 h, developed, and visualized by orcinol–H_2_SO_4_ reagent or fluorescence. SG indicates Sterylglucoside and GlcCer indicates glycosylceramide, *a* and *b* indicate hydroxylated and nonhydroxylated cerebroside respectively

A previous study indicated that 40.8–45.8 % of the fed ^3^H-labelled sphingosine portion of glycosylceramide purified from beef brain is recovered in faeces (Nilsson 1969). However, since koji glycosylceramide differs from bovine glycosylceramide in structure, the lipid profile of the faeces from mice fed with koji glycosylceramide was analyzed in order to investigate its metabolic fate during passage through the intestine. No clear band of glycosylceramide was detected (Fig. 2a), and ceramide or sphingoid bases were also not detected (data not shown) in the faeces. Considering that koji apparently contains only glycosylceramide, these results suggest that ingested koji glycosylceramide is metabolized or absorbed in the intestine either by intestinal enzymes or intestinal microbes.

### Koji glycosylceramide is not degraded by intestinal extract

The above results inspired us to hypothesize that koji glycosylceramide is digested in the upper intestine by intestinal enzymes. To prove this hypothesis, intestines were recovered from mice, homogenized, and incubated with koji glycosylceramide. Lipid profiles of koji glycosylceramide treated with the intestinal extract and untreated koji glycosylceramide were not significantly different (Fig. 2b). On the contrary, the intestinal enzymes had the ability to digest NBD-C12-ceramide (Fig. 2c). These results indicate that intestinal enzymes have the ability to degrade ceramide but not koji glycosylceramide; thus, koji glycosylceramide reaches the lower intestine where it is then metabolized by intestinal microbial flora.

### Identification of *Blautia coccoides* as the intestinal microbe that increases in number upon ingestion of koji glycosylceramide

**Fig. 3:**
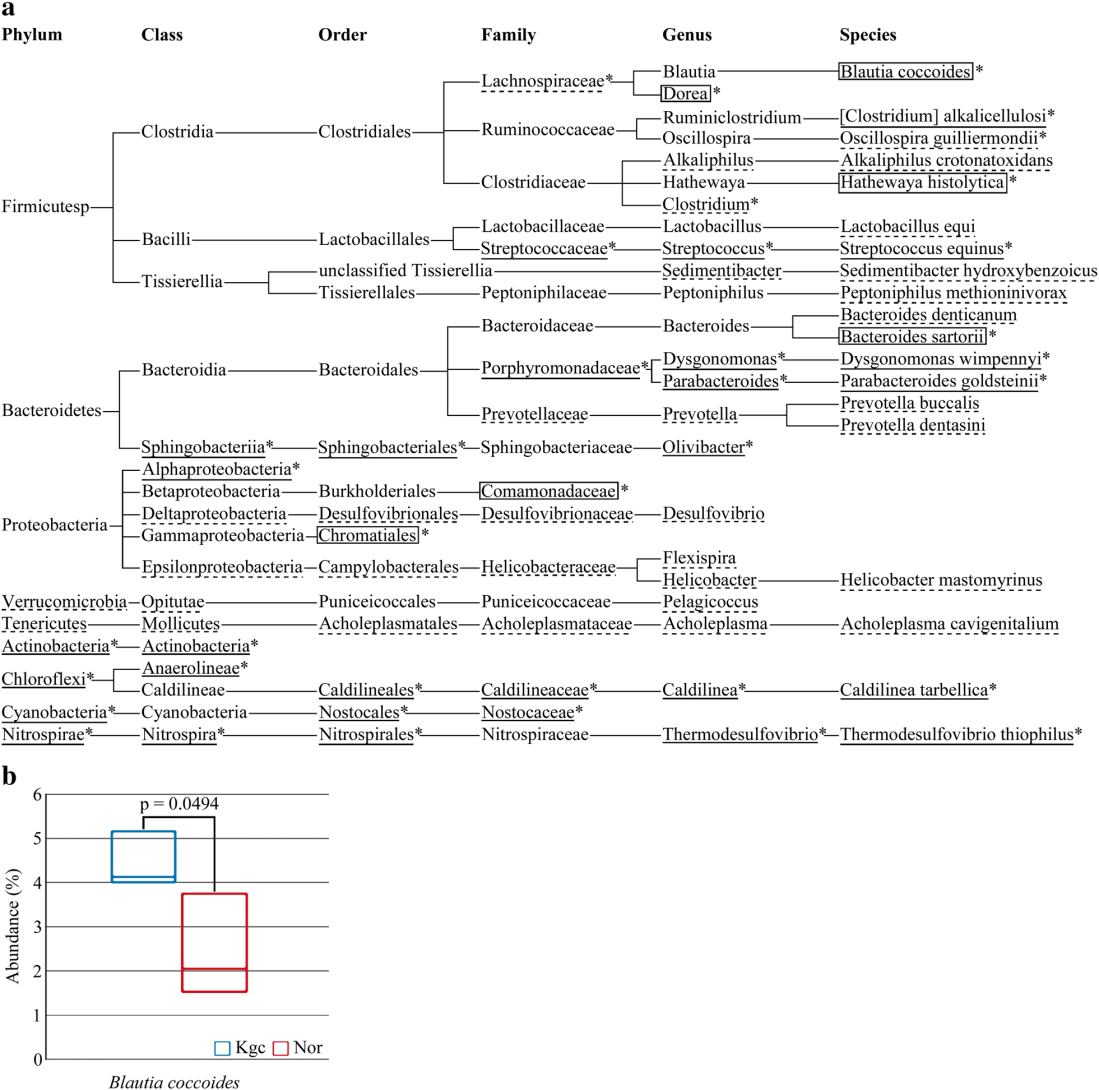
Analysis of intestinal microbial flora of mice fed with koji glycosylceramide. **a** Cluster tree of microbes increased or decreased in the feces of mice fed with koji glycosylceramide. The *underline* indicates microbes whose read values were significantly increased (*p* < 0.05). The *broken underline* indicates microbes whose percentage values were significantly increased (*p* < 0.05). The *square* indicates microbes whose read values and percentage values were significantly increased. *Stars* indicate microbes which were larger in Kgc than in Nor. **b** Box plot representing the relative abundance of the genera (*Blautia coccoides*) enriched in the feces of koji glycosylceramide-fed mice. Mice were fed with koji glycosylceramide for 1 week. The feces were recovered, and freeze-dried. Genomic DNA was extracted and purified from the feces. The V3–V4 variable region of 16S rRNA was amplified using PCR and sequenced using Miseq, Illumina. Data were analyzed by MiSeq Reported 16S metagenomics system. *p* value indicates one-tailed unpaired Student’s t-test under symmetry conditions (n = 3)

The results above suggested that koji glycosylceramide reaches the lower intestine and affects intestinal microbial flora. Therefore, in order to analyze the intestinal microbial flora, genomic DNA was extracted from the faeces of koji glycosylceramide-fed or non-fed mice. It turned out that *B. coccoides*, *Dorea*, *Clostridium alkalicellulosi*, *Hathewaya histolytica*, *Bacteroides sartorii*, *Dysgonomonas*, *Dysgonomonas wimpennyi*, *Alphaproteobacteria*, *Chromatiales*, and *Chloroflexi* significantly increased in response to the addition of koji glycosylceramide (*p* < 0.05, Fig. 3a). *B. coccoides* is a strict anaerobe, which is often found in the mammalian intestine (Park et al. 2013).

### In vitro stimulation of the growth of *Blautia coccoides* by koji glycosylceramide

**Fig. 4:**
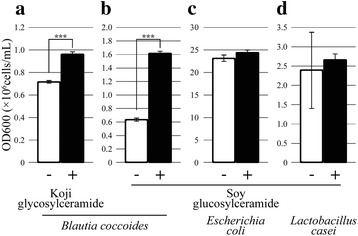
In vitro analysis of the bacterial growth-stimulating effect of koji glycosylceramide. Purified glycosylceramide was added to bacterial cultures and growth (OD_600_) was measured after 24 h of culture. The results are the mean of triplicate independent experiments with standard errors. The statistical significance of differences between averages was assessed by the unpaired one-tailed Student’s t-test (***p < 0.001). **a** *B. coccoides* incubated with or without 4 μg/μL koji glycosyleeramide. **b** *B. coccoides* incubated with or without 4 μg/μL soybean glucosylceramide. **c** *L. casei* incubated with or without 4 μg/μL soybean glucosylceramide. **d** *E. coli* incubated with or without 4 μg/μL soybean glucosylceramide. Detailed materials and methods are described in the text

The above feeding experiments were based on glycosylceramide purified from koji. However, the purification of glycosylceramide was not complete (Fig. 1b), thus it was not clear if pure glycosylceramide would stimulate the growth of these bacteria. However, preparation of 2.8 g of pure koji glycosylceramide was difficult. Therefore, investigation of stimulation of bacterial growth by koji glycosylceramide was conducted in vitro. Since *B. coccoides* is attracting greater attention because of its effects on health, we focused on *B. coccoides*. Bacteria were incubated with purified glycosylceramide and the growth was monitored. Consistent with our hypothesis, the growth of *B. coccoides* was stimulated with either koji glycosylceramide (Fig. 4a) or soy glucosylceramide (Fig. 4b). On the contrary, glycosylceramide did not affect the growth of general intestinal microbes such as *Escherichia coli* (Fig. 4c) or *Lactobacillus casei* (Fig. 4d). These results support our hypothesis that koji glycosylceramide specifically stimulates the growth of *B. coccoides* in the intestine.

## Discussion

Although the Japanese people have eaten Japanese cuisine on a national level for centuries and presently have one of the longest healthy life spans in the world, the relationship between Japanese cuisine and Japanese longevity has remained obscure. In this study, we elucidate that koji glycosylceramide modulates intestinal microbial flora, specifically by increasing *B. coccoides*. This mechanism might be a new connection between Japanese cuisine and Japanese longevity.

*Blautia coccoides* is one of the major intestinal microbes often found in human faecal samples (Park et al. 2013). It is a strict anaerobe and gram-positive bacteria recently reclassified from *Clostridium* (Liu et al. 2008). There are growing reports of the relationship between decreased levels of *B. coccoides* and disease, such as cirrhosis and hepatic encephalopathy (Bajaj et al. 2012), colorectal cancer (Chen et al. 2012), intestinal inflammation (Jenq et al. 2012), breast cancer (Mabrok et al. 2012), type I diabetes (Murri et al. 2013), irritable bowel syndrome (Rajilić-Stojanović et al. 2011), acute diarrhoea, and idiopathic inflammatory bowel disease (Suchodolski et al. 2012). Furthermore, several reports suggest that increasing the ratio of *B. coccoides* in the intestine might be beneficial for health. Indeed, diets high in resistant starch and arabinoxylan increase the ratio of *B. coccoides* in the intestinal microbial flora (Nielsen et al. 2014), as do omega 3 fatty acids (Myles et al. 2014). In addition, *B. coccoides* decreases the NF-κB activity in Caco-2 cells (Lakhdari et al. 2011; Jenkins et al. 2002). *B. coccoides* does not evoke an inflammatory response in mononuclear cells (Tuovinen et al. 2013). Therefore, there is evidence that intestinal *B. coccoides* contributes to health.

The modification of intestinal *B. coccoides* by Japanese cuisine and its effect on health is of significant concern. Several studies point out that changes in the intestinal population levels of the genus *Blautia* are dependent on the diet, age or nationality. Indeed, the genus *Blautia* was most frequently found in the faeces of Japanese people (Nishijima et al. 2016) relative to other nations in the world, supporting the hypothesis that Japanese cuisine containing koji increases *B. coccoides* in the intestine. Intestinal population levels of *Blautia* in children with type I diabetes are significantly higher than those in healthy children (Murri et al. 2013). In addition, the population level of *Blautia* is decreased in the intestines of cirrhotic patients (Kakiyama et al. 2013), as well as in elderly people relative to young people (Kurakawa et al. 2015). Together with the knowledge attained in this study, it is likely that Japanese people have increased levels of intestinal *B. coccoides* through consumption of koji glycosylceramide contained in the Japanese cuisine, contributing to the health status of the Japanese people (Symolon et al. 2004; Fujiwara et al. 2011; Yazama et al. 2015). This hypothesis needs further study and verification.

The practical effect of koji glycosylceramide in the daily intake of Japanese traditional cuisine can be inferred from this study. In this study, mice (19.8 g weight) ate 20.4 g per week, which corresponds to 0.147–0.294 (g/day)/(g body weight). Since koji glycosylceramide was present in the feed at 0.2–1 % w/w, this corresponds to 0.71–1.43 g/day for a 60 kg human [human equivalent doses (Reagan-Shaw et al. 2008)]. Considering that Japanese people eat 5–100 g koji per day and koji contains 0.5–3 mg/g glycosylceramide, Japanese people intake 0.0025–0.3 g koji glycosylceramide per day. Therefore, the doses used in this study can be considered sufficiently effective in humans.

Previous studies provide sufficient evidence that dietary glycosylceramide is digested and absorbed in the intestine (Nilsson 1969; Schmelz et al. 1994). Therefore, since intestinal enzymes cannot degrade glycosylceramide (Fig. 2b), intestinal microbes are considered to have the ability to degrade glycosylceramide to ceramide. Consistent with this hypothesis, intestinal *Blautia glucerasei* was shown to degrade glucosylceramide to ceramide (Furuya et al. 2010). From these facts, it appears that these microbes degrade koji glycosylceramide to ceramide, and the resulting sugar moiety and ceramide are metabolized to fatty acids and sphingoid bases, which are then absorbed in the intestine (Nilsson 1969). This is a significant target of the next study.

Several studies reported that dietary soy or rice bran glucosylceramide reduces cancers such as colon cancer and head and neck cancer (Symolon et al. 2004; Fujiwara et al. 2011; Yazama et al. 2015). In addition, plant-origin glucosylceramide suppresses bowel inflammation (Arai et al. 2015). Furthermore, dietary glucosylceramide improves skin function (Kawada et al. 2013; Duan et al. 2012; Tsuji et al. 2006; Miyanishi et al. 2005). Alteration of intestinal microbiota, as found in this study, might be the cause of these observed phenomena.

## Conclusion

In conclusion, we have elucidated that the Japanese dietary fungus koji *A. oryzae* functions as a prebiotic, since glycosylceramide contained in koji (rice fermented with *Aspergillus oryzae*) increases *B. coccoides* in the intestine. This knowledge might be a novel link between Japanese cuisine and Japanese longevity.
