# Erratum to: Japanese traditional dietary fungus koji *Aspergillus oryzae* functions as a prebiotic for *Blautia coccoides* through glycosylceramide: Japanese dietary fungus koji is a new prebiotic

**DOI:** 10.1186/s40064-016-3539-9

**Published:** 2016-10-25

**Authors:** Hiroshi Hamajima, Haruka Matsunaga, Ayami Fujikawa, Tomoya Sato, Susumu Mitsutake, Teruyoshi Yanagita, Koji Nagao, Jiro Nakayama, Hiroshi Kitagaki

**Affiliations:** 1 Department of Environmental Science, Faculty of Agriculture, Saga University, Honjo-cho, Saga City, Saga Japan; 2 Department of Applied Biochemistry and Food Science, Faculty of Agriculture, Saga University, Honjo-cho, Saga City, Saga Japan; 3 Faculty of Health and Nutrition Science, Nishikyushu University, Ozaki, Kanzaki-cho, Kanzaki City, Saga Japan; 4 Department of Bioscience and Biotechnology, Faculty of Agriculture, Kyushu University, Hakozaki, Higashi-ku, Fukuoka, Japan

## Erratum to: SpringerPlus (2016) 5:1321 DOI 10.1186/s40064-016-2950-6

Upon publication, the authors noticed that in the original version of the article (Hamajima et al. 2016), there were two errors.

1. In line 8 of the legend of Fig. 4, “glycosyleeramide” should read “glycosylceramide” (as it is in every other instance of the word).
2. In Table 2, the words “soluble fraction” and “insoluble fraction” should be interchanged in the column “Chloroform”.

**Table 2:** Purification summary of glycosylceramide from koji

	Koji lipid	Chloroform		Acetone	
		Insoluble fraction	Soluble fraction	Insoluble fraction	Soluble fraction
Weight of total recovery		0.39 g ± 0.01	0.59 g ± 0.01	0.25 g ± 0.04	0.24 g ± 0.06
Weight of glycosylceramide	24.49 mg ± 2.49	0.17 mg ± 0.02	22.24 mg ± 0.84	17.80 mg ± 1.63	3.64 mg ± 2.36
Purification rate of glycosylceramide	2.46 % ± 0.21	0.04 % ± 0.00	3.79 % ± 0.18	7.28 % ± 0.59	1.41 % ± 0.56

Summary of glycosylceramide purification from 1 g of koji lipid. Glycosylceramide was purified from koji by chloroform–acetone fractionation

The results are expressed as mean values ± standard deviation of three independent experiments

Please see the corrected Table 2 below:

These errors have now been corrected in this erratum. We, the publishers, apologise that these errors were missed and for any inconvenience caused by this.
